# PIS-YOLO: Real-Time Detection for Medical Mask Specification in an Edge Device

**DOI:** 10.1155/2022/6170245

**Published:** 2022-11-17

**Authors:** Zuopeng Zhao, Xiaofeng Liu, Kai Hao, Tianci Zheng, Junjie Xu, Shuya Cui

**Affiliations:** ^1^School of Computer Science and Technology & Mine Digitization Engineering Research Center of Ministry of Education of the People's Republic of China, China University of Mining and Technology, Xuzhou 221116, China; ^2^School of Computer Science and Technology, China University of Mining and Technology, Xuzhou 221116, China

## Abstract

Wearing masks in a crowded environment can reduce the risk of infection; however, wearing nonstandard cloud does not have a good protective effect on the virus, which makes it necessary to monitor the wearing of masks in real time. You only look once (YOLO) series models are widely used in various edge devices. The existing YOLOv5s method meets the requirements of inference time, but it is slightly deficient in terms of accuracy due to its generality. Considering the characteristics of our driver medical mask dataset, a position insensitive loss which is cloud extract shared area feature in different categories and half deformable convolution net methods with cloud concern noteworthy features were introduced into YOLOv5s to improve accuracy, with an increase of 6.7% mean average in @.5 (mAP@.5) and 8.3% in mAP@.5:.95 for our dataset. To ensure that our method can be applied in a real scenario, TensorRT and CUDA were introduced to reduce the inference time in two edge devices (Jetson TX2 and Jetson Nano) and one desktop device, whose inference time was faster than that of previous methods.

## 1. Introduction

With the emergence of SARS-Cov-2 variants, the mean value of R0, an indicator reflecting infectivity, has evolved from initial 2.79 to 5.08 [1], indicating a considerable increase in its infectivity, and it has become necessary for people to wear medical masks correctly in public places. However, breathing discomfort caused by wearing the medical masks results in a nonstandard wear phenomenon, that is, the exposure of the wearer's nose or mouth, and this phenomenon intensifies the spread of the virus, particularly in crowded public places with poor ventilation, such as public transport. The detection of the quality of medical masks in these cases is particularly important, and detection may be performed for both passengers and supervisors.

Object detection based on deep learning, which exceeds traditional methods in many areas [2, 3], has been widely used. Lie et al. detected the wear condition of medical mask target locate person face and the categories of wear condition. We divided the categories into mask, no mask, and incorrect wear, which can promote the protection of passenger themselves and reduce infection risk. The current object detector can be divided into two categories. On the one hand, two-stage object detection is based on the propose-classfy approach, such as the family of R-CNN [4–6]. The two-stage algorithm has high accuracy but slow detection speed. On the other hand, one-stage object detection algorithms treat the detection task as a regression problem, such as SDD [7] and YOLO series [8–12]. Due to its excellent detection speed and small volume of model, it is more widely used for the edge device.

In addition to algorithm selection, selecting a proper device for model to work normally is important. Patrikar et al. conducted a detailed review on anomaly detection with video surveillance [13] and considered anomaly detection to be a time-critical application of computer vision. In this study, the transfer of video stream from a mobile terminal to the central server and the subsequent analysis of the results was not feasible in terms of time. Additionally, the costs of video data and transmission remain unaffordable. Thus, we used a remote edge device, which saved considerable network resources, for videos. However, the energy consumption, volume, cost, and other factors of edge equipment limit edge device wide application and deployment. With the continuous development of hardware resources, the computing performance of Jetson AGX Xavier for mobile detection has become more enhanced than that of GTX 1080TI, which is often used on a desktop to train models. Although its inference cost is considerably high, several edge devices with high computational ability make real-time detection feasible.

Although in our previous study [14] the medical mask wearing condition could be detected using the you only look once (YOLOv4) [8] algorithm, researchers did not use any model acceleration tools to make full use of the current device and only employed python, an interpretive language with low performance, which limits its use in real-world applications. Moreover, YOLOv4 is inferior in performance to YOLOv5 [9]. Therefore, in this study, to target these defects, we mainly focused on the following:

1. Introduce position insensitive loss (PIS) into YOLOv5 to improve detection quality, especially the category with few samples
2. Introduce the half deformable convolution net (HDCN) module into YOLOv5 to extract features located in half part of the feature map, while maintaining the computing cost
3. Compare and evaluate multiple networks in multiple edge devices with model acceleration tools

## 2. Related Work

Presently, YOLO series are the most popular one-stage object detector. In view of its excellent performance, it is used for real-time detection of various tasks. The same goes for mask detection. Su et al. proposed an algorithm, which integrates transfer learning and efficient-Yolov3 [15] based on RMFD [16] dataset and MAFA [17] dataset, for face mask detection task. Liu Againan used YOLOv3 [18] to complete the task. Loey et al. [19] employed YOLOv2 [10] and ResNet [20] for medical face mask detection. Liu et al. [21] used YOLOv3 and simple CNAPs to improve the classification performance of real-time mask detection. Cao et al. [22] proposed a mask detection namely MaskHunter based on YOLOv4. Kumar et al. [23] improved tiny YOLOv4 with spatial pyramid pooling (SPP) module and used the tiny YOLOv4-SPP network for face mask detection. Although all of them achieved high performance in mask detection, the performance of YOLOv2-v4 was inferior to that of YOLOv5 [9].

Zhang et al. [24] determined whether a person was wearing a mask using VGG19 [25]; however, they ignored the category of incorrect wearing and the computing cost of VGG19 with large computation. Alguzo et al. [26] used multigraph convolutional networks (MGCNs) to accurately detect people wearing masks. This caused mask wearing detection to not be limited to CNN.

Zhang et al. used the convolutional neural network (CNN) to detect whether a driver was holding a phone [27] based SSD [7]; this scenario is similar to our task of detecting whether the driver is wearing a mask. Liu et al. conducted a survey of deep neural network architectures and their applications [28], which could facilitate our design of network architecture. Ahmad et al. unsupervised real-time anomaly detection for streaming data [29], which provided novel solutions for sample shortage and imbalance.

Algorithms are important for mobile devices and hardware. MobileNet [30] proposed by Google is a classic lightweight network. MobileNet is usually combined with (single-shot multibox detector) SSD [7] for object detection. MobileNet-SSD is one of the main demos provided by some hardware manufacturers or deep learning inference frameworks (such as PaddleLite [31] and RKNN [32], which is a model inference tool for Rockchip products) for object detection. It is usually used to compare and evaluate hardware or inference framework benchmarks, but its accuracy and speed are insufficient in current context. Although two-stage regions with CNN features (R-CNN) series [4–6] network can achieve a high accuracy, its detection speed is not ideal for edge devices. With the continuous development and optimisation of various models in terms of the accuracy and speed, the YOLO series [8–12] models have gradually gained popularity in practical industrial applications.

YOLOv5 remains an active project. On 12 October 2021, YOLOv5-v6 was published; thus, although both of them are YOLOv5, the structure of YOLOv5 described in our paper is different from that described in [3]. YOLOv5 uses two scaling factors to control the depth and width of the network, resulting in different YOLOv5 structures that meet the trade-off between the calculated cost and accuracy. From YOLOv5n, YOLOv5s, YOLOv5m, and YOLOv5l to YOLOv5x, both the accuracy and computing cost have continuously increased. YOLOv5n, the abbreviation of YOLOv5 nano, was proposed in YOLOv5-v6. Similar to YOLOv3, YOLOv5 can be used to predict four position parameters, namely, *t*_*x*_, *t*_*y*_, *t*_*w*_, and *t*_*h*_, which are closely related to our proposed method.

**Figure 1:**
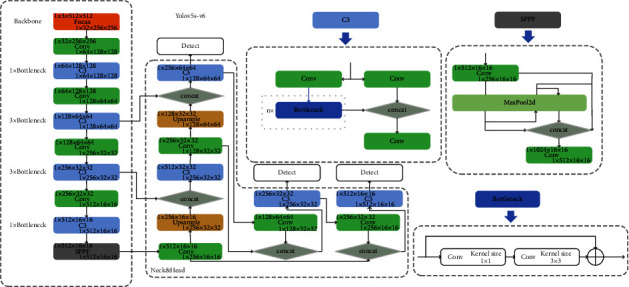
Version of the Yolov5-v6 structure and specific dimensional transformations on Yolov5s. The upper left and lower right corner represent the input and output dimensions of the current module, respectively.

Figure 1 shows the component of YOLOv5-v6, and our research is based on this. C3 is an abbreviation for the CSP bottleneck with three convolution layers, which omits one convolution module, one batch normalisation layer, and one activation function on the basis of the CSP bottleneck, reducing the number of parameters. C3 also takes an extra parameter to control the number of times the bottleneck module repeats, as shown in the left side of Figure 1; the larger is the number of repetitions, the stronger is the ability of the network to extract depth features. The right top of Figure 1 is the 6^th^ version proposed spatial pyramid pooling fast (SPPF), which is faster than existing SPP [33].

Above all, although approximately half of the previous mask detection is based on YOLO studies, and YOLO is widely used in various applications, it still have to consider some points. On the one hand, they use a lower version of YOLO with lower performance, and on the other hand, they are limited to using relevant deep learning frameworks to measure network speed, without considering practical applications such as in edge devices. Our research based model use recent versions and related acceleration measures to reduce the detection time and improve the accuracy simultaneously.

## 3. Materials and Methods

### 3.1. Datasets

**Figure 2:**
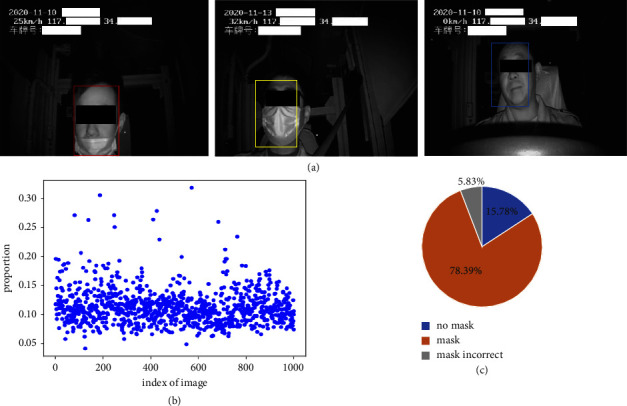
(a) Partial sample of the driver mask dataset. (b) Object proportion for the driver mask dataset. (c) Category proportion for the driver mask dataset.

We first introduced the Driver Mask dataset [14] with the categories of mask, no mask, and incorrect mask. A total of 2360 images with the pixel of 1280 × 720 have been divided into train set and test set. The Driver Mask dataset consists of 2360 images with three categories, and its resolution is 1280 × 720. We randomly selected 1888 images (80%) as the train set, while the rest 472 images (20%) were used as the test set. Our previous study described our collected driver medical mask dataset, but it did not describe the bounding box distribution and proportion of categories.  Figure 2(a) shows our dataset. We divided it into three categories (from left to right) such as incorrect mask, mask, and no mask. We randomly extracted 1000 objects to measure the proportion of the object to the entire image from Figure 2(b). Different from our previous research, we removed some incorrectly marked samples and the samples from the MAFA dataset. We inferred that the smallest object occupied approximately 5% pixel in the entire image, which indicated that there were few small objects in COCO [34] definitions (objects with an area of <32^2^ were categorised as small objects, objects with an area of 32^2^–96^2^ were categorised as medium objects, and the rest were categorised as large objects).

**Figure 3:**
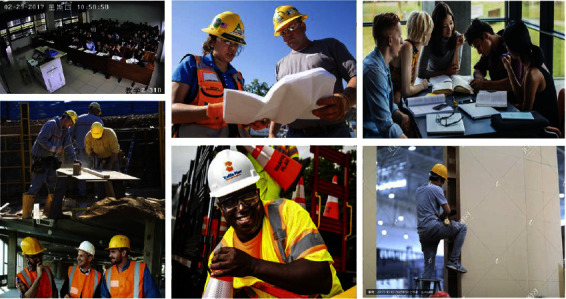
Partial sample of the safety helmet dataset contains various scenarios.

In order to prove that our method also has a good performance in other detection tasks, we introduced the Safety Helmet [35] dataset with the categories of person and hat. A total of 6056 images with different pixel have been divided into train set and val set. The train set of Safety Helmet contains 4845(80%) images, and the test set contains 1211(20%) images, including construction site photos, surveillance video images of a university, and ordinary scene photos, and the detection task was to correctly identify whether the person in the image is wearing a helmet.  Figure 3 shows a partial sample of the dataset.

### 3.2. Proposed Methods

According to the distribution of our datasets, we extracted two facts: (1) all the three categories shared the same position features and (2) the features that the network should distinguish are limited in the half bottom of anchors. Inspired by these two facts, we proposed PIS loss to make the network learn extra position features, and the HDCN to make the network learn the features in the half bottom of the bounding box rather than the whole feature map.

#### 3.2.1. PIS Loss

Similar to the previous YOLO series, YOLOv5 also simultaneously predicts the object classes and position. In addition to two direct explicit predictions for a user, the predicted IOU or object parameter cloud makes the model rapidly converge. To make full use of object position features of all classes rather than those of one particular category, we proposed PIS loss to allow the network focus on position features, which could improve the mAP of category with few samples in the premise of all categories that shared the uniform position features. In equal 1, *α*, *β*, and *γ* are hyperparameters, and *n*_*i*_ represents the number of iterations of model forward. The right part of equal 1 represents the detailed composition of *n*_*i*_. *nb* is the constant, which is the number of batch size; symbols epoch and *i* are variables, which represent the index of the current epoch and index in this epoch.

Thus, if the *n*_*i*_ is odd, the current iteration deprecates position loss and retain class and object loss; otherwise, the current iteration retains all losses. We used fine-grained *n*_*i*_ rather than *b*_*i*_, which represents the index of the current batch size, because it can accelerate model convergence. Because the index starts with zero, the model first learns complete features. Both the hyperparameter and constant are fixed during training:

(1) $$\begin{array}{c} L=\left\{\begin{array}{cc} \alpha L_{\text{class}}+\beta L_{\text{obj}}+\gamma L_{b\text{box}}\text{,} & n_{i}\;is\;even\text{,}\\ \alpha L_{\text{class}}+\beta L_{\text{obj}}\text{,} & n_{i}\;is\;odd\text{,} \end{array}\right.\\ n_{i}=i+nb*\text{epoch.} \end{array}$$

Equal 1 proposed PIS Loss.

#### 3.2.2. HDCN

The top half of the bounding box (Figure 2(a)) is occupied by people's eyes and eyebrows; however, all of these features are not related to the medical mask detection task in categories; that is, the mask detection task does not require the position feature of the whole face but only the desired part of the face for classification.

**Figure 4:**
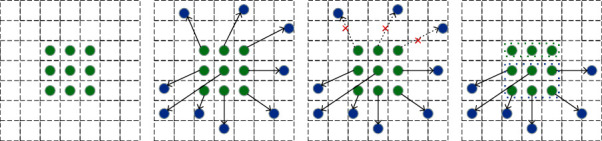
(a) Ordinary convolution operator. (b) Deformable convolution net. (c) Deformable convolution net with restrained offset. (d) Our proposed HDCN.

Convolution operator samples the fixed position of the input feature map. To focus on the features located in the half bottom of the feature map, rather than the whole feature map, a feasible solution is that a deformable convolution [36] operator should accept the complete input tensor and restrain the offset predicted by an extra convolution net. However, this method may require high computational cost. Another possible solution is to cut the input tensor into two sections as shown in Figure 4: the top half part can be handled by the convolution operator, and the half bottom part can be handled with the deformable net. This slicing tensor methodology is applied in the focus module in the 5^th^ version of YOLOv5, which reduces the computational cost and improves the model accuracy.

**Figure 5:**
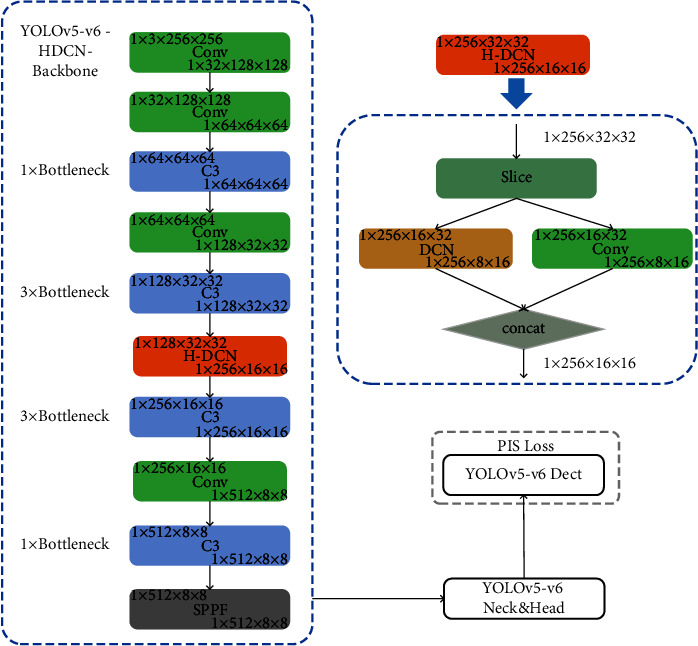
Overall architecture: proposed YOLOv5s-v6-HDCN with PIS loss.

The position of the HDCN module in the network must be studied. If this module is used to extract the deep feature of the model with a small feature map, the ability of distinguishing feature in similar datasets will be interference; that is, the smaller the feature HDCN handles, the less are the advantages of HDCN. In addition, it is seen that the HDCN could reduce computational cost when compared with ordinary DCN.  Figure 5 presents a detailed structure of our proposed YOLOv5-HDCN-PIS.

## 4. Experiment

### 4.1. Experimental Set

In this study, the experimental platform was an Intel Core I7 10700KF processor, with 32 GB memory, NVIDIA GeForce RTX 3090 24 GB memory with PyTorch 1.8.1, Python 3.8, and the Ubuntu 20.04 operating system. Because the official DCNv2 does not work on high version PyTorch, which supports RTX30 series devices, we used MMCV [37] to support this operator. This configuration was also appropriate for measuring the inference time, as discussed in Section 5.

For the environment mentioned in Section 3.1, we trained our models with different datasets and batch sizes. The size of all the input images was fixed to 224^2^, and mosaic data enhancement was used to improve model performance. To accelerate training, we employed early stop; that is, when 100 epochs were attained without improvement, training got stopped. All the training epochs were fixed to 300, and the symbol epoch*∗* was used to indicate the actual epoch trained by the model. In addition, to improve model accuracy, we used the official supportedYOLOv5-v6 pretrained weight.

### 4.2. Experimental Results

Table 1 presents the experiment results. We extracted the original YOLOv5-v6 and our proposed YOLOv5s-v6-HDCN with PIS loss. The ↑ symbol suggests that our proposed method, in relation to the existing methods, presents the improvement of mAP@.5 and mAP@.5:.95 indicators (Table 1). On the model Yolov5s-v6, the incorrect mask category shows the lowest accuracy for both mAP@.5 and mAP@.5:.95 indicators because the incorrect mask category has the least samples. In addition, from the number of actual training epochs, we can infer that the original method is easier to converge, and our proposed module requires more time to training.

The highest improvement in accuracy of 18.6% and 17.8% is observed for the incorrect mask category. The lowest improvement in accuracy of 1.4% and 4% is obtained for the mask category. We can conclude that the lesser the samples in a category, the more the accuracy of that category cloud can be improved through PIS loss. After HDCN module and PIS loss, the accuracy of the incorrect mask category was close to that of the other two categories. The accuracy increased for all the categories largely depends on categories with the least samples. Besides the experimental results shown in Table 1, YOLOv5s-v6 takes 7018216 parameters, and Yolov5s-v6-HDCN takes 7266973 parameters.

### 4.3. Ablation Experiment

In this section, targeting both our methods can improve the accuracy to detect whether the driver is wearing a medical mask. We mainly discuss the effects of our proposed PIS and HDCN based on ordinary YOLOv5s-v6 (Table 2). Because mAP@.5:.95 is less than mAP@.5 in YOLOv5s-v6, after the application of the two methods, the improved accuracy for mAP@.5:.95 is higher than that for mAP@.5. The highest improvement in accuracy is obtained for the incorrect mask class in the model YOLOv5s-v6-HDCN, and the mAP@.5 indicator and mAP@.5:.95 improved by 10.5% and 12%, respectively. For YOLOv5s-v6 with PIS loss, the two accuracies are 6.4% and 12.8%.

In addition, to confirm that our proposed PIS loss works on other datasets, we extracted the safety helmet dataset, whose categories are more balanced compared with our datasets, which showed 1.3% and 1% improvement for mAP@.5 and mAP@.5:.95, respectively. Experiments showed that PIS loss may improve accuracy for a part of the datasets whose categories shared the same position feature but different class features.

## 5. Model Deployment

The model deployment target made full use of the current device and accelerated the inference time. Some deep learning frameworks, such as PyTorch, TensorFlow, Paddle, and Caffee, provide both training and inferencing ability, but all of them export the interface using python. In spite of the native implementations of performance-sensitive invocation interfaces, which can be C++, CUDA, or platform-specific libraries, this cross-language invocation inhibits the compiler optimisation strategy, and the python virtual machine invokes the nonpython code, causing an additional performance penalty and resulting in the inference time being inferior to that of pure C++ implementations. In addition, some model acceleration tools launched by hardware manufacturers or inference frameworks can automatically optimise the model structure by graph optimisation, etc., in their products. However, some of them support python. Although python interfaces are provided in part projects, they may be suitable only for faster validation. In the end, the latest supported features or latest fixed exceptions are often implemented using C++.

It is difficult to deploy a simple complicated model trained with a high-performance device on an edge device. On the one hand, different deep learning frameworks export different format training results; on the other hand, the ARM architecture is often used in edge devices because of power consumption, volume, etc., and some frameworks or software work well on an X86 platform, such as Anaconda, which is one of the most popular data science platforms that does not fit or partially fits the Advanced RISC Machine (ARM) platform and limits the correct build environment for deployment. In addition, the central processing unit (CPU) is suitable for general computing tasks, and a GPU or other specific devices, such as Huawei NPU and Rockchip NPU, are highly suitable for the forwarding of a neural network. However, the adaptation of operators is a crucial problem. For PaddleLite, a majority of the operators are supported on CPUs, followed by GPUs (non-Nvidia devices are usually indirectly supported through the OpenCL interface), and the specific devices with minimal operators support the remaining operators. These difficulties limit the high-performance deployment of the model and require close coordination between software engineers and hardware engineers.

### 5.1. Model Conversion

**Figure 6:**
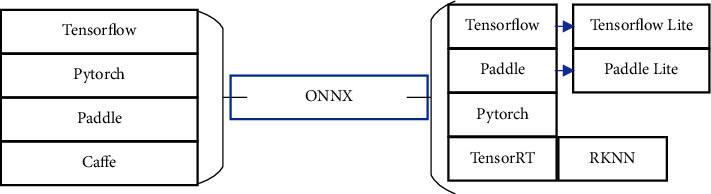
Models trained by part deep learning frameworks can be converted into ONNX, and TensorFlow Lite and PaddleLite models must first be converted to TensorFlow or Paddle models. TensorRT, PaddleLite, TensorFlow Lite, and RKNN are all aimed at the inference and not the training.

Model conversion is a crucial step of deployment; it translates the model exported by the deep learning framework mentioned above into a format suitable for the target device or platform. Furthermore, torch2trt is a tool only for a PyTorch model converted to TensorRT; the alternative method is to convert these models to the ONNX format indirectly and then converts ONNX to the target device.  Figure 6 shows a sample in which a part of frameworks is converted in one direction or two directions.

Although a model is converted to the ONNX format successfully, whether it can be converted to the target platform remains unknown, and its supported operators depend on its maintainers rather than on us, making it difficult to customise our own operators. Furthermore, the use of an automatic model conversion tool may cause some problems, such as unsupported operators or inconsistencies in the converted model's prediction result and the actual prediction result, leading to debugging difficulties. To improve the reliability and maintainability of the model and reduce the debugging cost, Wang-Xinyu maintained the TensorRTx [38] project on GitHub, which is used to develop various models, such as YOLOv5, YOLOv3, and YOLOv3-SPP, by manually calling the TensorRT API to construct the network structure.

Our proposed HDCN module highly depends on DCN. Because there is no existing DCN module in the TensorRT API list, we extracted the implementation of deformable convolution with TensorRT by using MMCV and integrated a source code into the project with CMake. We developed the HDCN operator plugin and integrated it into the backbone of YOLOv5.

### 5.2. Deployment Set

In addition to the training environment mentioned in Section 3.1, we introduced two types of edge devices: Jetson TX2 and Jetson Nano. Both of them are flashed into JetPack 4.6 (with CUDA10.2, OpenCV 4, TensorRT 8, cuDNN 8). Our program developed with CMake can run directly on each edge device. However, the environment that supports our algorithm implementation by PyTorch is difficult to construct. With docker, we obtained the PyTorch images based on NVIDIA Linux4Tegra (L4T) from the Nvidia GPU Cloud (NGC). Setting up a deployment environment on an edge device with the ARM platform is time consuming. Because of the performance of the device, we ran the container and installed MMCV through source code compilation and the residual dependency by pip on Jetson TX2. Then, we moved the container to Jetson Nano, which reduced the environment development time and maintained environment consistency. We took the float precision for both the platform and environment.

We mainly measured inference time in various devices. The size of all the input images was fixed to 224^2^, and all the max batch sizes were fixed to 1. In addition to the two edge devices, we introduced the RTX 3090 for training the model. We measured not only the forward time but also the preprocessing time (such as image enhancement or format conversion) and postprocessing time (such as NMS or mapping detection results to the original image); all of these times were measured in milliseconds. Both preprocessing and postprocessing did not include read images from the disk or write images to the disk, which can avoid the interference of the storage device type on the experimental results. The ACC column with a tick denotes that the current line's result was handled using accelerate tools, such as TensorRT and CUDA, and the ACC column without a tick indicates that the current line's result was handled with PyTorch and other python tools, whose detail configuration is discussed in Section 3.1. We extracted approximately 200 images from the driver mask dataset as the test set; we took the average of preprocess and postprocess time for different devices and models but with the same model accelerate strategy and showed the inference time separately.

### 5.3. Deployment Result

As inferred from Section 4.2, although the parameter of Yolov5s-HDCN is slightly more than Yolov5s-HDCN, its inference time is not proportional to parameters in some situations. Different from the accuracy, the inference time measured in devices is susceptible to current temperature, CPU or GPU scheduling policy, and the other application process the device may execute, which results in randomness. Moreover, the inference time is affected by program quality and the program interface call mode. As the convolution operator is one of the most widely used operators, it has undergone considerable optimisation; however, our coding ability limits the full use of current devices.

Table 3 presents that the preprocess time with acceleration can become 2–5 times faster compared with python implementation; the postprocess time with acceleration can become 55–153 times faster compared with python implementation; and the forward time with acceleration can become 3–10 times faster compared with python implementation. In edge devices, the whole time can become three times faster with acceleration compared with python implementation. The most significant improvement is seen in the postprocess time because the NMS in postprocess is implemented with pure python in the PyTorch environment, which is slower than implementation by C++. The max FPS of the Yolov5s-v6 model on Jetson TX2, Jetson Nano, and RTX 3090 are 96, 64, and 1779, respectively, and the max FPS of the Yolov5s-HDCN model on JetsonTX2, Jetson Nano, and RTX 3090 are 102, 64, and 1639, respectively. The forward time requires the highest time in all the devices and models. Copying the tensor from the host memory to device memory and copying the forward result from the device memory to host memory can be considerably time consuming. Although the large GPU memory implied that this device could handle multiple images simultaneously, setting the batch size to 1 corresponds to the application of actual scenarios, such as real-time surveillance video analysis, which limits its throughput.

**Figure 7:**
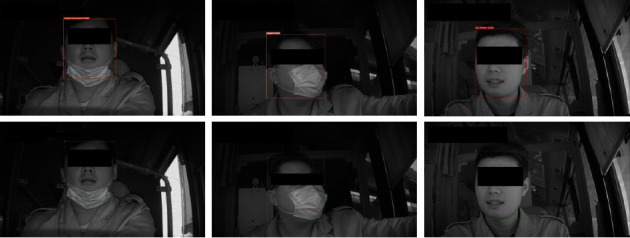
Detection result with PyTorch implementation and TensorRT implementation. We manually removed all the sensitive information after an image was processed by these frameworks.

The first row of Figure 7 shows the detection result in the case of PyTorch; the last row of Figure 7 presents the detection result in the case of accelerated by TensorRT and CUDA. The three columns present the three categories of our Driver Medical Mask dataset, which from left to right are incorrect mask, mask, and no mask. The images suggest after acceleration, the detection accuracy did not suffer large deviation.

## 6. Conclusions

In our research, deep learning technology was applied for medical mask detection. Based on YOLOv5s-v6, a highly accurate driver medical mask detection method with the PIS loss and HDCN modules, which fully consider the characteristics of our datasets, was proposed, resulting in an increase of 6.7% in mAP@.5 and 8.3% in mAP@.5:.95 for our Driver Medical dataset with a small loss of speed. In addition, PIS loss was introduced into public Safety Helmet dataset with shared location features among various categories, which resulted in an increase of 1.3% in mAP@.5 and 1% in mAP@.5:.95. If the introduced HDCN module cloud cannot be afforded, the PIS loss can result in an increase of 2% in mAP@.5 and 4.9 in mAP@.5:.95 in the Driver Mask dataset.

In addition, to make our algorithm suitable for real world application, we introduced Jetson Nano and Jetson TX2 as edge devices. The model accelerated tools to decrease inference time and increase throughput in the deployment environment, which resulted in a three times faster inference time than the time in the development environment developed with docker. Thus, a practicable model was successfully run on the two devices, which could contribute to mask detection tasks.

## 7. Future Work

Due to the limitation of our coding ability, the HDCN operator can still be improved to reduce the inference time. We will optimise the operator to speed up the inference time of the model. The expression form of PIS loss can be optimised and freed from the even or odd notion. Our model can be encapsulated with a docker container, and the container size can be reduced. The use of models in numerous edge devices will be promoted in the future research.

## Figures and Tables

**Table 1:** Experiment results of the comparison between original YOLOv5-v6 and our proposed YOLOv5s-v6-HDCN with PIS loss on the driver mask dataset. “Epoch*∗*” denotes the real num of epoch using early stopping. “Epoch” is the num of epoch manual setting. “↑” is used to show the improvement rate over YOLOv5s-v6.

Model	Imgsz	Bs	Epoch	Epoch*∗*	Class	mAP@.5	mAP@.5:.95
YOLOv5s-v6	224	64	300	151	All	0.877	0.639
					No_mask	0.905	0.631
					Mask	0.992	0.726
					Mask_incorrect	0.734	0.56

Yolov5s-v6-HDCN-PIS	224	64	300	242	All	0.944 ↑6.7%	0.722 ↑8.3%
					No_mask	0.919 ↑1.4%	0.671 ↑4.0%
					Mask	0.994 ↑0.2%	0.756 ↑3.0%
					Mask_incorrect	0.920 ↑18.6%	0.738 ↑17.8%

**Table 2:** Ablation experiment on the driver mask dataset and safety helmet dataset. “Epoch*∗*” denotes the real num of epoch using early stopping. “Epoch” is the num of epoch manual setting. “↑” is used to show the improvement rate over YOLOv5s-v6.

Model	Dataset	Imgsz	Bs	Epoch	Epoch*∗*	Class	mAP@.5	mAP@.5:.95
Yolov5s-v6-hdcn	driver_mask	224	64	300	182	All	0.910 ↑3.3%	0.686 ↑4.7%
						No_mask	0.897 ↓0.8%	0.638 ↑0.7%
						Mask	0.994 ↑0.2%	0.740 ↑1.4%
						Mask_incorrect	0.839 ↑10.5%	0.680 ↑12.0%
Yolov5s-v6-pis		224	64	300	217	All	0.897 ↑2.0%	0.688 ↑4.9%
						No_mask	0.899 ↓0.6%	0.651 ↑2.0%
						Mask	0.993 ↑0.1%	0.744 ↑1.8%
						Mask_incorrect	0.798 ↑6.4%	0.668 ↑12.8%
Yolov5s-v6	Safety_helmet	224	512	300	300	All	0.691	0.432
Yolov5s-v6-pis		224	512	300	300	All	0.704 ↑1.3%	0.442 ↑1.0%

**Table 3:** Inference time on multiplatform. “ACC” denotes the inference whether to use accelerate tools.

Model	ACC	Img size	Device											
			Jetson Tx2				Jetson Nano				RTX 3090			
			Preprocess	Forward	Postprocess	FPS	Preprocess	Forward	Postprocess	FPS	Preprocess	Forward	Postprocess	FPS
Yolov5s-v6	√	224^2^	0.268	10.049	0.061	96	0.313	15.311	0.055	64	0.036	0.517	0.009	1779
Yolov5s-HDCN	√			9.484		102		15.328		64		0.565		1639
Yolov5s-v6	×		1.456	31.980	3.564	27	1.592	46.765	8.435	18	0.081	5.896	0.501	154
Yolov5s-HDCN	×			30.519		28		46.625		18		5.999		150

## Data Availability

Code will be available from https://github.com/entropyfeng/yolov5-hdcn.

## References

[1] Liu Y., Gayle A. A., Wilder-Smith A., Rocklov J. (2020). The reproductive number of COVID-19 is higher compared to SARS coronavirus. *Journal of Travel Medicine*.

[2] Guo L., Wang D., Li L., Feng J. (2020). Accurate and fast single shot multibox detector. *IET Computer Vision*.

[3] Yao J., Qi J., Zhang J., Shao H., Yang J., Li X. (2021). A real-time detection algorithm for kiwifruit defects based on YOLOv5. *Electronics*.

[4] Ren S., He K., Girshick R., Sun J., Faster R.-C. N. N. (2017). Faster R-CNN: towards real-time object detection with region proposal networks. *IEEE Transactions on Pattern Analysis and Machine Intelligence*.

[5] Girshick R. Ieee. Fast R-CNN.

[6] Girshick R., Donahue J., Darrell T., Malik J. Ieee. Rich feature hierarchies for accurate object detection and semantic segmentation.

[7] Liu W., Anguelov D., Erhan D. (2016). SSD: single shot MultiBox detector. *Computer Vision – ECCV 2016*.

[8] Bochkovskiy A., Wang C. Y., Liao H. Y. M. (2020). YOLOv4: optimal speed and accuracy of object detection. https://arxiv.org/abs/2004.10934.

[9] Github (2021). Yolov5. https://github.com/ultralytics/yolov5.

[10] Redmon J., Farhadi A. (2017). YOLO9000: better, faster, stronger. *2017 IEEE Conference on Computer Vision and Pattern Recognition (CVPR)*.

[11] Redmon J., Divvala S., Girshick R., Farhadi A. Ieee. You only look once: unified, real-time object detection.

[12] Redmon J., Farhadi A. (2018). YOLOv3: an incremental improvement. https://arxiv.org/abs/1804.02767.

[13] Patrikar D. R., Rajram Parate M. (2021). Anomaly detection using edge computing in video surveillance system: review. https://arxiv.org/abs/2107.02778.

[14] Zhao Z., Hao K., Ma X. (2021). SAI-YOLO: a lightweight network for real-time detection of driver mask-wearing specification on resource-constrained devices. *Computational Intelligence and Neuroscience*.

[15] Su X., Gao M., Ren J., Li Y., Dong M., Liu X. (2022). Face mask detection and classification via deep transfer learning. *Multimedia Tools and Applications*.

[16] Wang Z., Wang G., Huang B. (2020). Masked face recognition dataset and application. https://arxiv.org/abs/2003.09093.

[17] Ge S., Li J., Ye Q., Luo Z. Ieee. Detecting masked faces in the wild with LLE-CNNs.

[18] Liu S., Agaian S. S. COVID-19 face mask detection in a crowd using multi-model based on YOLOv3 and hand-crafted features.

[19] Loey M., Manogaran G., Taha M. H. N., Khalifa N. E. M. (2021). Fighting against COVID-19: a novel deep learning model based on YOLO-v2 with ResNet-50 for medical face mask detection. *Sustainable Cities and Society*.

[20] He K., Zhang X., Ren S., Sun J. Deep Residual Learning for Image Recognition.

[21] Liu R., Ren Z. Application of Yolo on mask detection task.

[22] Cao Z., Shao M., Xu L., Mu S., Qu H (2020). MaskHunter: real time object detection of face masks during the COVID‐19 pandemic. *IET Image Processing*.

[23] Kumar A., Kalia A., Sharma A. (2021). A hybrid tiny YOLO v4-SPP module based improved face mask detection vision system[J]. *Journal of Ambient Intelligence and Humanized Computing*.

[24] Zhang E. Ieee. A REAL-TIME deep transfer learning model for facial mask detection.

[25] Simonyan K., Zisserman A. (2014). Very deep convolutional networks for large-scale image recognition. *Computer Science*.

[26] Alguzo A., Alzu’bi A., Albalas F. Masked face detection using multi-graph convolutional networks.

[27] Zhao Z., Zhang Z., Xu X., Xu Y., Yan H., Zhang L. (2020). A lightweight object detection network for real-time detection of driver handheld call on embedded devices. *Computational Intelligence and Neuroscience*.

[28] Liu W., Wang Z., Liu X., Zeng N., Liu Y., Alsaadi F. E. (2017). A survey of deep neural network architectures and their applications. *Neurocomputing*.

[29] Ahmad S., Lavin A., Purdy S., Agha Z. (2017). Unsupervised real-time anomaly detection for streaming data. *Neurocomputing*.

[30] Howard A. G., Zhu M., Chen B. (2017). Efficient convolutional neural networks for mobile vision applications. https://arxiv.org/abs/1704.04861.

[31] Github (2021). PaddleLite. https://github.com/PaddlePaddle/Paddle-Lite.

[32] Github (2021). Rknn. https://github.com/rockchip-linux/rknn-toolkit.

[33] He K., Zhang X., Ren S., Sun J. (2014). Spatial pyramid pooling in deep convolutional networks for visual recognition. *Computer Vision – ECCV 2014*.

[34] Lin T.-Y., Maire M., Belongie S. (2014). Microsoft COCO: common objects in context. *Computer Vision – ECCV 2014*.

[35] Yanxishe (2022). Safety HelmetDataset. https://god.yanxishe.com/12.

[36] Dai J., Qi H., Xiong Y. Ieee. Deformable convolutional networks.

[37] Github (2021). Mmcv. https://github.com/open-mmlab/mmcv.

[38] Github (2021). Tensorrtx. https://github.com/wang-xinyu/tensorrtx.

